# An Optimized Injectable Hydrogel Scaffold Supports Human Dental Pulp Stem Cell Viability and Spreading

**DOI:** 10.1155/2016/7363579

**Published:** 2016-05-16

**Authors:** T. D. Jones, A. Kefi, S. Sun, M. Cho, S. B. Alapati

**Affiliations:** ^1^Bioengineering, University of Illinois at Chicago, Chicago, IL 60612-7212, USA; ^2^Endodontics, University of Illinois at Chicago, Chicago, IL 60612-7212, USA

## Abstract

*Introduction*. HyStem-C*™* is a commercially available injectable hydrogel composed of polyethylene glycol diacrylate (PEGDA), hyaluronan (HA), and gelatin (Gn). These components can be mechanically tuned to enhance cell viability and spreading.* Methods*. The concentration of PEGDA with an added disulfide bond (PEGSSDA) was varied from 0.5 to 8.0% (w/v) to determine the optimal concentration for injectable clinical application. We evaluated the cell viability of human dental pulp stem cells (hDPSCs) embedded in 2% (w/v) PEGSSDA-HA-Gn hydrogels. Volume ratios of HA : Gn from 100 : 0 to 25 : 75 were varied to encourage hDPSC spreading. Fibronectin (Fn) was added to our model to determine the effect of extracellular matrix protein concentration on hDPSC behavior.* Results*. Our preliminary data suggests that the hydrogel gelation time decreased as the PEGSSDA cross-linker concentration increased. The PEGSSDA-HA-Gn was biocompatible with hDPSCs, and increased ratios of HA : Gn enhanced cell viability for 14 days. Additionally, cell proliferation with added fibronectin increased significantly over time at concentrations of 1.0 and 10.0 *μ*g/mL in PEGDA-HA-Gn hydrogels, while cell spreading significantly increased at Fn concentrations of 0.1 *μ*g/mL.* Conclusions*. This study demonstrates that PEG-based injectable hydrogels maintain hDPSC viability and facilitate cell spreading, mainly in the presence of extracellular matrix (ECM) proteins.

## 1. Introduction

Endodontics is a specialized dental field concerned with treating infected and traumatized dental tissues. During embryonic tooth development, the cranial neural crest gives rise to developing dental epithelium. Ectomesenchymal cells originate from the dental epithelium and form the dental papilla, an aggregate of differentiated ectomesenchymal cells called odontoblasts. During embryogenesis these highly specialized cells secrete primary dentin, a mineralized tissue surrounding dental pulp. Dental pulp is the soft tissue inside of the tooth core that contains nerve and blood vessels subject to thermal, chemical, mechanical, and bacterial insults.

Dental caries formation is the most prevalent disease affecting human teeth and may result in early tooth loss if left untreated [1]. Root canal therapy (RCT) is an endodontic procedure replacing infected or injured pulp tissue with a biologically inert material to resolve pain and control infection [2–5]. Reinfection of the pulp chamber due to bacterial invasion and susceptibility of tooth fracture may result in failed RCTs that require retreatment [6–8]. The successful design of a biological substitute for dental-pulp tissue will likely require a tissue engineering approach designed to restore, maintain, or improve dental-pulp tissue function applying both regenerative endodontics and engineering principles. Recent advances in these two fields support the regeneration potential of pulp tissue using human dental pulp stem cells (hDPSCs) embedded in bioactive scaffolds [9, 10].

This cell source holds considerable promise for dental engineering applications as they have been shown to differentiate into both avascular mineralizing tissues and the vasculature necessary for adequate nutrient exchange [4]. Despite recent progress in stem cell biology for translational clinical approaches, the scaffolds designed for dental pulp tissue engineering applications must be temporally and spatially optimized to set efficiently in complex geometries such as the root canal [9] while simultaneously facilitating cell attachment and spreading [11].

Cell attachment to a scaffold is likely required for the terminal differentiation of hDPSC into functional dentin-secreting odontoblasts [9]. Several groups have reported successful hDPSC attachment on natural or synthetic scaffolds such as collagen or poly(D,L-lactide-co-glycolide) (PLGA), respectively [12–15], but both scaffold types are limiting when considered independently. Natural scaffolds are biocompatible and biodegradable but may be immunogenic and weak [12]. Although synthetic scaffolds may be mechanically tuned and processed into desired shapes, they do not contain environmental cues for cell attachment found in the natural extracellular matrix (ECM) [10]. In addition to cell attachment, cell spreading on a substrate can greatly influence cell proliferation and death. Although cell-ECM interactions such as mediation with adhesive proteins have been well studied, the effect of early hDPSC spreading both on the surface of and embedded in scaffolds is less established. Interestingly, material properties have been shown to independently influence cell spreading.

Recently, it has been shown that “scaffoldless” constructs engineered from hDPSCs promote pulp vascularization [8], but the high cell density requirement, extensive material handling, and lack of characterization of the cells responsible for a regenerated dentin-pulp complex-like structure do not outweigh the clinical translation of more established injectable scaffold models [16] such as hydrogels. Injectable hydrogels combine the benefit of natural and synthetic scaffolds [17] and are practical for syringe applications. To our knowledge, no commercially available injectable hydrogel has been reported specifically for dental pulp tissue engineering applications.

HyStem-C is a thiol-reactive hyaluronan-based (HA) hydrogel that is cross-linked with synthetic polyethylene glycol diacrylate (PEGDA 3400) and thiol-reactive gelatin (Gn). It has been shown to facilitate tissue regeneration in various organ systems including the regeneration of subchondral bone [18]. The molecular weight of the PEG-based cross-linker and concentrations of HA and Gn can be manipulated to mimic desired tissue characteristics [18]. Another modular feature of the HyStem-C system is the ability to add or substitute ECM proteins with the Gn to better mirror target organ architecture.

In this paper, we chemically altered the HyStem-C components to investigate hDPSC biocompatibility and potential for syringe injection into the root canal lumen. We substituted PEGSSDA 8400 in place of PEGDA 3400 and assessed the hydrogel gelation time as a function of cross-linker concentration to increase the mechanical strength. SS indicates the presence of a disulfide bond that enables nonenzymatic cell recovery [18]. Next, we varied the ratio of  HA : Gn to optimize cell spreading. A third goal of this paper was to determine the effect of fibronectin, a native dental pulp EMC protein, on hDPSC spreading. The overall goal of this study was to test the hypothesis that a PEGDA-HA based hydrogel combined with gelatin and fibronectin can support hDPSC viability and spreading using an in vitro injectable three-dimensional (3D) cell delivery strategy.

## 2. Materials and Methods

### 2.1. Cell Culture

Human DPSCs were kindly gifted by Dr. Songtao Shi (University of Southern California) and expanded in vitro as previously reported by Gronthos et al. [19]. Briefly, cells between 3rd and 5th passages were grown in single-cell suspensions and seeded in 25 cm^2^ culture flasks containing *α*-MEM medium (Invitrogen, Grand Island, NY, USA) supplemented with 20% fetal bovine serum (FBS), 2 mM L-glutamine, 100 mM L-ascorbic acid 2-phosphate, 100 U/mL penicillin, and 100 *μ*g/mL streptomycin and incubated at 37°C in 5% CO_2_.

### 2.2. Evaluation of Gelation Time as a Function of PEGSSDA Concentration

HyStem-C (BioTime, Inc., Alameda, CA) is a hyaluronan-based hydrogel kit containing a thiolated cross-linker (PEGDA molecular weight 3400), thiol-reactive gelatin (denatured collagen), and thiol-reactive hyaluronan. Thiolated PEGSSDA molecular weight ~8400 (Glycosan Biosystems, Inc., Alameda, CA) was used to cross-link the HA and Gn. HA and Gn were dissolved in phosphate-buffered saline (PBS) pH 7.4 to give 1.0% (w/v) solutions. Next, the HA and Gn solutions were mixed in a 1 : 1 (v/v) ratio. To determine the optimal gelation time of PEGSSDA (Glycosan Biosystems) as a function of concentration, the (w/v) of PEGSSDA was varied from 0.5% to 8%. The following concentrations were prepared in triplicate: 0.5%, 1.0%, 2.0%, 4.0%, and 8.0%. Each concentration was diluted in a 1 : 1 (v/v) ratio of hyaluronic acid : gelatin. The final aqueous solution was passed through a 20-gauge needle to model clinical application. Gelation time as a function of concentration was analyzed using nonlinear regression modeling SPSS software (Chicago, IL, USA). Solution-gel transitions were determined by the test tube transition inversion method at room temperature [20].

### 2.3. 3D Modeling of hDPSC

Human DPSCs were embedded in 100 *μ*L of PEGSSDA-HA-Gn hydrogels at a density of 3.0 × 10^4^ cells per cell culture tissue insert (Millipore, Billerica, MA, USA) in a 24-well plate. Cell culture insert dimensions were 13 mm in diameter and 10.5 mm in height, with a pore size of 8.0 *μ*m. Briefly, a cell pellet was suspended in a 1 : 1 (v/v) ratio of HA : Gn. One volume of 2% (w/v) PEGSSDA was added to the HA + Gn cell slurry in a 1 : 4 volume ratio. The cell-embedded hydrogels were allowed to gel for one hour at 37°C in 5% CO_2_ in the tissue culture inserts. Following gelation, 1.0 mL of cell culture medium was added to each insert-containing well. The medium was changed every other day.

### 2.4. Variation of Volume Ratio of Thiolated Hyaluronan : Thiolated Gelatin

To encourage cell spreading, the volume ratio of thiolated HA : thiolated Gn (Glycosan Biosystems, Inc., Alameda CA) was varied. A cell pellet (3.0 × 10^4^ human dental pulp stem cells) was suspended in each of four volume ratios of 1 : 0, 3 : 1, 1 : 1, and 1 : 3 corresponding to the ratio of HA : Gn. Next one volume of 2.0% (w/v) PEGSSDA was added to four volumes of each prepared HA : Gn + cell sample. All components were dissolved in sterile PBS pH 7.4 and allowed to gel at room temperature for one hour. Experiments were performed in triplicate wells for each volume ratio mixture. The cells were analyzed for cell viability using a live/dead in vitro cell viability assay (Molecular Probes, Eugene, OR) at days 7 and 14 as per the manufacturer's instructions.

### 2.5. Addition of Fibronectin to PEGDA-HA-Gn Hydrogels

To determine the effect of fibronectin on cell viability, proliferation, and spreading, purified human fibronectin (Millipore, Billerica, MA, USA) in phosphate buffer saline pH 7.4 was varied from 0.1 to 10 *μ*g/mL and added to 1 : 1 (v/v) ratios of thiolated HA and Gn solutions. PEGDA 3400 (2.0% w/v) was added to the HA-Gn-Fn mixture in a 1 : 4 volume ratio to form 200 *μ*L hydrogels in cell culture inserts of a 24-well plate. Positive control (PEGDA-HA-Gn) and negative control (PEGDA-HA-Fn) hydrogels were prepared in parallel. HDPSCs were serum-starved for 24 hours and seeded on the hydrogel surface at a density of 1 × 10^4^ cells per 200 *μ*L of hydrogel solution. The serum-free media were replaced with regular growth media after 24 hours. Samples were analyzed for cell proliferation, viability, and spreading at days 1 and 4 in triplicate. The cells were analyzed for cell viability using a viability assay (Molecular Probes, Eugene, OR) at days 1 and 4 as per the manufacturer's instructions.

### 2.6. Analysis of hDPSC Proliferation on PEGDA-HA-Gn-Fn Hydrogels

Human dental pulp stem cell proliferation as a function of varying fibronectin concentration was assessed using a cell proliferation colorimetric assay (Roche, Indianapolis, IN, USA). At days 1 and 4, both the PEGDA-HA-Gn-Fn experimental and positive and negative control hydrogels seeded with 1 × 10^4^ hDPSCs were transferred to fresh wells of a 24-well plate. The relative hDPSC density was assessed following the addition of 20 *μ*L of WST-1 (2-(4-iodophenyl)-3-(4-nitrophenyl)-5-(2,4-disulfophenyl)-2H-tetrazolium, monosodium salt) reagent per well to achieve a 1 : 10 final concentration. The samples were incubated for three hours at 37°C in 5% CO_2_ to allow the color conversion of the tetrazolium salt due to mitochondrial activity. The absorbance was measured using a microplate reader at 450 nm (BioTek, Winooski, VT, USA). Experiments were performed in triplicate wells per concentration at both days 1 and 4.

### 2.7. Fluorescent Microscopy

Cell viability was observed using fluorescent microscopy. All samples treated for cell viability (Molecular Probes, Eugene, OR) were visualized with an E-800 Eclipse Nikon fluorescent microscope with a 20x objective lens and a 16-bit charge-coupled device camera (Photometrics, Tuscan, AZ, USA). Images were pseudocolored with MetaMorph imaging software (Molecular Devices, Sunnyvale, CA, USA). The Image J software bundle (National Institutes of Health, Bethesda, MD, USA) was used to quantify the effect of fibronectin concentration on hDPSC spreading. Red, green, and blue (RGB) images were split into constituent channels, and images were converted to 8-bit greyscale images. The “threshold” command was applied to classify the hDPSC morphology based on cell surface area.

### 2.8. Analysis of the Effect of Fibronectin Concentration on hDPSC Spreading

Cell spreading at days 1 and 4 was quantified following the classification of hDPSC morphology into three distinct categories: round, partially spread, and fully spread. Postprocessing imaging using Image J software was used to determine the cell surface area in pixels^2^. Cell rounding or spreading was calculated as (average number of round or spread cells/40 cells per field of view) × 100%. Four random fields per hydrogel were chosen for each sample and 10 cells were selected at a time to obtain 40 cells. Three independent samples were tested at both time points.

### 2.9. Statistical Analysis

Statistical analysis for all triplicate data was analyzed using the one-way ANOVA followed by Tukey's post hoc test (SPSS, Chicago, IL, USA). A *p* value < 0.05 was considered significant.

## 3. Results

### 3.1. Quantification of Gelation Time as a Function of PEGSSDA Concentration

**Figure 1:**
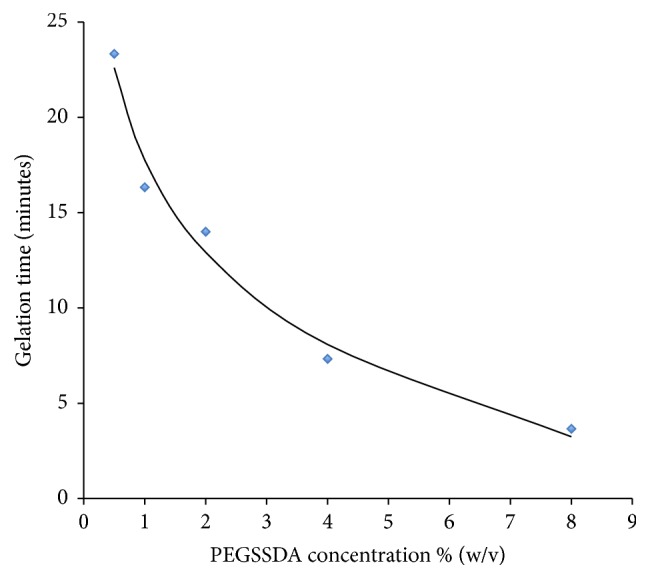
Gelation time as a function of PEGSSDA concentration fit to a nonlinear logarithmic regression model. Diamonds represent experimental data. The curved line is theoretical data.

Figure 1 shows the average gelation time of the PEGSSDA-HN-Gn hydrogels as a function of varying PEGSSDA concentration. The data represents the average gelation times of experiments conducted in triplicate and fit to a nonlinear logarithmic regression model. Diamonds denote experimental data, while the curved line represents theoretical data. The gelation time decreased as the percentage of (w/v) PEGSSDA concentration increased from 0.5% to 8.0%. The residual sum of squares (RSS) method was used to measure the discrepancy between the experimental and theoretical models and was calculated as 4.482.

### 3.2. Variation of Volume Ratio of Thiolated Hyaluronan : Thiolated Gelatin

**Figure 2:**
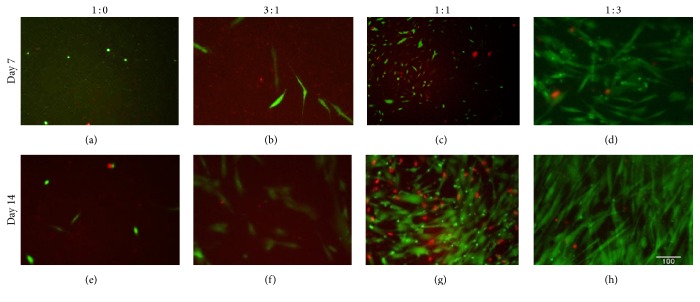
Immunofluorescence results for live/dead staining of human dental pulp stem cells embedded in 2.0% (w/v) PEGSSDA and varying (v/v) ratios of HA : GN. Green = live cells. Red = dead cells. Magnification = 20x. Scale bar = 100 *μ*m.

**Figure 3:**
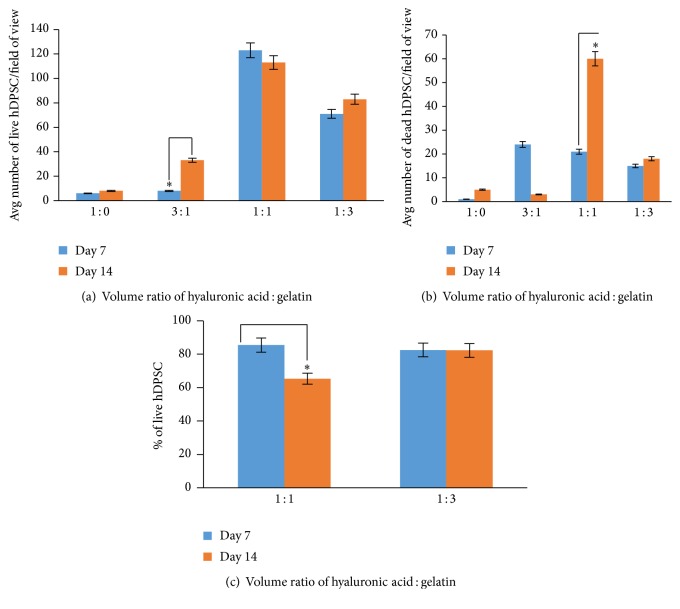
(a-b) The average number of live and dead hDPSC per field of view as a function of (v/v) ratio of HA : GN. (c) The percentage of live hDPSC in the 1 : 1 and 1 : 3 (v/v) ratios of HA : GN. ^*∗*^ *p* < 0.05.

Figure 2 shows the hDPSC live/dead viability staining results of varied ratios of HA : Gn while maintaining a 2.0% (w/v) concentration of PEGSSDA. In general, the hDPSCs were more spindle-shaped in the 1 : 1 and 1 : 3 volume ratios of HA : Gn compared to the 1 : 0 and 3 : 1 HA : Gn volume ratios. The average number of live and dead cells per microscopic field of view is shown in Figures 3(a) and 3(b). Based on the limited number of cells in the field of view for the 1 : 0 and 3 : 1 HA : Gn ratios, we calculated the percentage of viable cells based on HA : Gn ratios for the 1 : 1 and 1 : 3 mixtures only (Figure 3(c)). The percentage of viable cells embedded in the 1 : 1 ratio of HA : Gn significantly decreased between days 7 and 14 (Figure 3(c)).

### 3.3. Analysis of hDPSC Proliferation on PEGDA-HA-Gn-Fn Hydrogels

**Figure 4:**
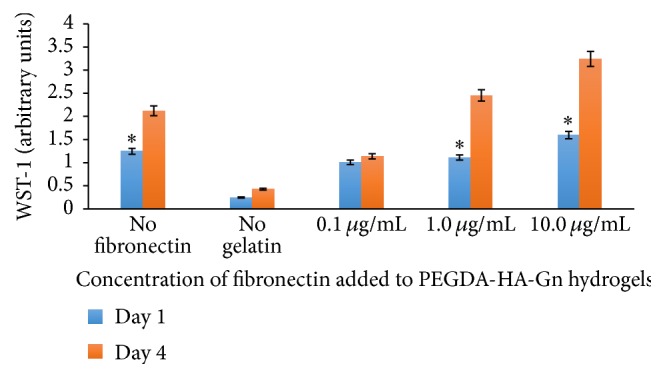
Proliferation of hDPSC in PEGDA-HA modified hydrogels with different concentrations of fibronectin. *n* = 3 samples. WST-1 was used to determine the relative cell densities at days 1 and 4. ^*∗*^ *p* < 0.05.

The results of the WST-1 assay are shown in Figure 4. The hDPSCs survived in all PEGDA based formulations. There was a significant increase from days 1 to 4 in the PEGDA-HA-Gn hydrogels with 1.0 *μ*g/mL and 10.0 *μ*g/mL of added human fibronectin. The positive control hydrogels lacking fibronectin also showed a significant increase in the relative human dental pulp stem cell density at day 4.

### 3.4. Human Dental Pulp Stem Cell Viability on PEG-Based Hydrogels

**Figure 5:**
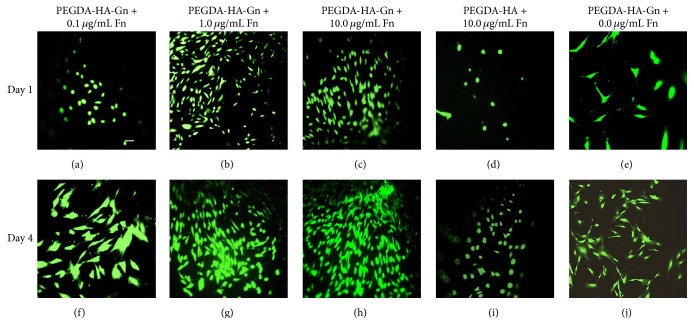
(a–j) Immunofluorescence staining results of human dental pulp stem cells seeded on the surface of polyethylene glycol diacrylate-based hydrogels. Data represents a sample of a random field of view from 4 random fields of view per hydrogel. Green = live cells. Magnification = 20x. Scale bar = 50 *μ*m.

We observed an increase in cell viability from days 1 to 4 in all PEGDA-HA-Gn hydrogels at all three concentrations of added fibronectin (see Figures 5(a)–5(c) and 5(f)–5(h)). Negative control samples without gelatin (PEGDA-HA) with 10.0 *μ*g/mL of added fibronectin appeared to maintain a round morphology (Figures 5(d) and 5(i)). The positive control samples without added fibronectin were more spindle-shaped with increased spacing between individual cells (Figures 5(e) and 5(j)).

### 3.5. Analysis of the Effect of Fibronectin Concentration on hDPSC Spreading

**Figure 6:**
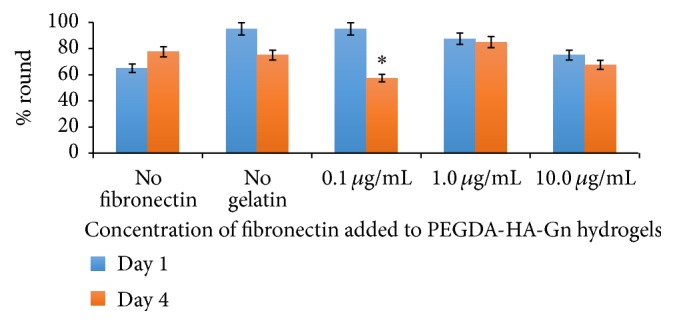
Effect of fibronectin concentration on hDPSC rounding. Data represents an average of 40 cells/field of view at 20x magnification. *n* = 3 samples. ^*∗*^ *p* < 0.05.

**Figure 7:**
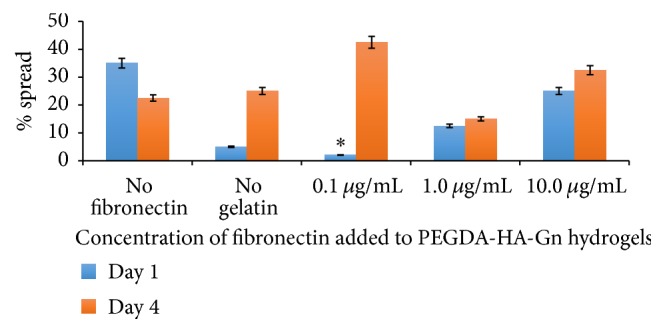
Effect of fibronectin concentration on hDPSC spreading. Data represents an average of 40 cells/field of view at 20x magnification. *n* = 3 samples. ^*∗*^ *p* < 0.05.

Table 1 shows the classification of hDPSC morphology based on the cell surface area range in pixels^2^. Three distinct categories were identified based on the cell morphology. The percentage of rounded cells at day 4 in hydrogel samples containing PEGDA-HA-Gn supplemented with 0.1 *μ*g/mL of fibronectin (Figure 6) was significantly less compared to day 1. This data corresponds to a significant increase in the percentage of partial and fully spread hDPSCs at the same hydrogel composition at day 4 (Figure 7).

## 4. Discussion

The present study builds on the dental tissue engineering triad model for pulp regeneration, combining a cell source, scaffold, and growth factors as possible alternatives to root canal therapy [21]. HDPSCs are an attractive cell source because they are multipotent and capable of differentiating into odontoblasts, vascular endothelial cells, fibroblasts, and neural cells [3]. Although cell differentiation was not a functional aim of this paper, we considered the potential of a single cell source that would maintain viability and spread in an injectable scaffold. Cell-based strategies for regenerative endodontics will require user-friendly cell delivery methods that are capable of gelation in pulp chambers [10].

Despite the potential of injectable scaffolds with self-assembling peptides, these hydrogels are limited by poor mechanical strength and lack of pore shape control [22]. To address material and fabrication limitations, we aimed to optimize hDPSC cell viability and spreading by embedding cells in a 3D commercially available injectable scaffold with tunable mechanical properties. We hypothesized that a PEGSSDA (mol wt 8400) cross-linker would be more biocompatible with hDPSCs compared with a PEGDA (mol wt 3400) due to greater mechanical strength. Theoretically, this increased mechanical strength is due to a higher cross-linking density in the PEGSSDA. As such, an early research goal was to determine the relationship between gelation time and the PEGSSDA used in our experiments.

There was an inverse relationship between the gelation time and concentration. Higher PEGSSDA concentrations resulted in lower gelation times. This data is consistent with findings from Gupte and Ma, who suggested that a dense hydrogel network is formed due to an increase in the number of cross-links [23]. Although our experimental findings can be considered as a predictive model for product gelation time for syringe applications, future studies should examine the contribution of the HA and gelatin independently as a function of gelation time to increase the model accuracy. Conducting these experiments may result in a lower residual sum of squares value.

To simulate clinical application, we used a 2% (w/v) PEGSSDA concentration to enable sufficient product gelation time of approximately 14 minutes following extrusion from a 20-gauge needle. After maintaining a constant 1% (w/v) of HA and Gn mixed in a 1 : 1 volume ratio with the PEGSSDA, we noted that hDPSCs were viable in the PEGSSDA-HA-Gn hydrogel but exhibited a rounded morphology rather than a flat spindle shape (data not shown). These results were consistent with a study comparing fibroblast morphology in a PEGDA-HA-Gn product [24]. We then varied the volume ratio of HA : Gn in an effort to increase hDPSC cell spreading.

For the first time, we report an optimal ratio of HA : Gn for cell delivery in an injectable PEG-based hydrogel scaffold. A 1 : 3 ratio of HA : Gn sustained cell viability in the PEGSSDA-HA-Gn scaffolds for up to 14 days in vitro. This could be due to increased gelatin content resulting in increased cell adhesion binding sites [25–27]. The 1 : 1 ratio of HA : Gn significantly increased the percentage of nonviable hDPSCs (data not shown) in the scaffold, which may be attributed to cell culture insert space limitations. Though this is not the aim of this study, PEGSSDA also enables nonenzymatic cell recovery from the hydrogel if desired [28].

The modular inclusion of cell adhesion proteins is a second benefit of using injectable tunable hydrogels for cellular encapsulation [29]. In concert with our long-term goal of fabricating a physiologically relevant hybrid extracellular matrix mimic, we seeded hDPSCs on the surface of PEGDA-HA-Gn scaffolds and varied the concentration of added fibronectin. We selected the PEGDA 3400 as a cross-linking agent for these experiments for two main reasons. Firstly, the viscoelastic properties of PEGDA-HA-Gn scaffolds have been well characterized by Ghosh et al. [30], reporting an elastic modulus of ~550 Pa. More importantly, human dental pulp is a soft tissue; thus we hypothesized that the PEGDA 3400 backbone would be biologically applicable to hDPSCs that were not biochemically induced to differentiate into harder mineralized tissues. These initial 2D experiments are an essential prerequisite to forthcoming 3D studies.

Harumi Miyagi et al. demonstrated that Fn showed the least variation in a study of ECM matrix protein expression in human hDPSC donors [31]. The cell-adhesion signaling cascades mediated by Fn binding allow cells to attach to the ECM and function. We hypothesized that adding Fn to our model would increase cell proliferation, viability, and spreading over time. Despite promising results with our earlier 1 : 3 (v/v) ratios of HA : Gn, we noted that the Fn contains binding domains for gelatin. To account for this, we designed our experiments to include a 1 : 1 volume ratio of HA : Gn. Data from the WST-1 absorbance values suggests that as the concentration of the Fn increases, the hDPSC proliferation increases. This data contrasts the effect of PuraMatrix*™* on hDPSC proliferation, which is a self-assembling injectable scaffold [9] that did not demonstrate a concentration dependent relationship.

Interestingly, the biological ligand density on a surface is a major factor that controls both cell attachment and spreading. Our data showed a significant increase in cell spreading based on the Fn concentration of 0.1 *μ*g/mL at day 4. This may have been due to the classification of “spreading” based on our methods. We acknowledge that Figure 7 includes cells that are classified as both partially and fully spread. Although the hDPSCs embedded in the PEGSSDA-HA-Gn hydrogels appeared to spread based on qualitative morphology, hDPSCs seeded on the surface of PEGDA-HA-Gn positive control hydrogels decreased in spreading after four days based on our classification. Thus fibronectin appears to contribute to hDPSC spreading. This hypothesis can be experimentally validated by conducting experiments that quantify the RGD ligand density required for cell spreading [32].

In sum, both collagen and fibronectin are expressed in native dental pulp, and it remains to be seen how the mutual integrin binding sites affect hDPSC fate in injectable hybrid scaffolds. The next step would be to determine how protein modified PEGDA-based injectable hydrogels influence embedded hDPSC attachment and integrin expression.

## 5. Conclusion

Our results provide new insight for the purposeful design of dental pulp tissue engineering applications. An injectable scaffold with tunable mechanical and biochemical components may facilitate targeted tissue mimicry.

## Figures and Tables

**Table 1:** Classification of hDPSC morphology in pixels^2^ based on cell surface area range.

Cell surface area range (pixels^2^)	Cell morphology
0–399	Round
400–1599	Partially spread
>1600	Fully spread
